# Aging and working memory modulate the ability to benefit from visible speech and iconic gestures during speech-in-noise comprehension

**DOI:** 10.1007/s00426-020-01363-8

**Published:** 2020-07-05

**Authors:** Louise Schubotz, Judith Holler, Linda Drijvers, Aslı Özyürek

**Affiliations:** 1 Max Planck Institute for Psycholinguistics, P.O. Box 310, 6500 AH Nijmegen, The Netherlands; 2 Donders Institute for Brain, Cognition, and Behaviour, P.O. Box 9010, 6500 GL Nijmegen, The Netherlands; 3 Centre for Language Studies, Radboud University Nijmegen, P.O. Box 9103, 6500 HD Nijmegen, The Netherlands

## Abstract

When comprehending speech-in-noise (SiN), younger and older adults benefit from seeing the speaker’s mouth, i.e. visible speech. Younger adults additionally benefit from manual iconic co-speech gestures. Here, we investigate to what extent younger and older adults benefit from perceiving both visual articulators while comprehending SiN, and whether this is modulated by working memory and inhibitory control. Twenty-eight younger and 28 older adults performed a word recognition task in three visual contexts: mouth blurred (speech-only), visible speech, or visible speech + iconic gesture. The speech signal was either clear or embedded in multitalker babble. Additionally, there were two visual-only conditions (visible speech, visible speech + gesture). Accuracy levels for both age groups were higher when both visual articulators were present compared to either one or none. However, older adults received a significantly smaller benefit than younger adults, although they performed equally well in speech-only and visual-only word recognition. Individual differences in verbal working memory and inhibitory control partly accounted for age-related performance differences. To conclude, perceiving iconic gestures in addition to visible speech improves younger and older adults’ comprehension of SiN. Yet, the ability to benefit from this additional visual information is modulated by age and verbal working memory. Future research will have to show whether these findings extend beyond the single word level.

**Electronic supplementary material:**

The online version of this article (10.1007/s00426-020-01363-8) contains supplementary material, which is available to authorized users.

## Introduction

In every-day listening situations, we frequently encounter speech embedded in noise, such as the sound of cars, music, or other people talking. Relative to younger adults, older adults’ language comprehension is often particularly compromised by such background noises (e.g. Dubno et al., 1984). However, the visual context in which speech sounds are perceived in face-to-face interactions, particularly the speaker’s mouth movements and manual gestures, may facilitate the comprehension of speech-in-noise (SiN). Both younger and older adults have been shown to benefit from visible speech, i.e. the articulatory movements of the mouth (including lips, teeth and tongue) (e.g. Sommers et al., 2005; Stevenson et al., 2015; Tye-Murray et al., 2010; 2016). Recent work has also demonstrated that younger adults’ perception of a degraded speech signal benefits from manual iconic co-speech gestures in addition to visible speech (Drijvers & Özyürek, 2017; Drijvers et al., 2018). Co-speech gestures are meaningful hand movements which form an integral component of the multimodal language people use in face-to-face settings (e.g. Bavelas & Chovil, 2000; Kendon, 2004; McNeill, 1992). Iconic gestures in particular can be used to indicate the size or shape of an object or to depict specific aspects of an action and thus to communicate relevant semantic information (McNeill, 1992). Whether older adults, too, can benefit from such gestures is currently unknown. The aim of the current study was to find out whether and to what extent older adults are able to make use of iconic co-speech gestures in addition to visible speech during SiN comprehension.

In investigating this question, we also consider whether hearing loss and differences in cognitive abilities play a role in this process. Both factors have been associated with the disproportionate disadvantage older adults experience due to background noises (e.g. Anderson et al., 2013; CHABA, 1988; Humes, 2002, 2007; Humes et al., 1994; Pichora-Fuller et al., 2017; see also Akeroyd, 2008). While age-related hearing loss has direct effects on central auditory processing, it also increases the cognitive resources needed for speech perception (Sommers & Phelps, 2016). Aging is frequently associated with declines in cognitive functioning, e.g. working memory (WM) or inhibitory mechanisms (Hasher, Lustig, & Zacks, 2007; Hasher & Zacks, 1988; Salthouse, 1991). In combination with hearing loss, this may further contribute to an overall decrease in resources available for cognitive operations like language comprehension or recall (e.g. Sommers & Phelps, 2016). Accounting for sensory and cognitive aging is thus crucial in the investigation of older adults’ comprehension of SiN and the potential benefit they receive from visual information.

Previous research suggests that perceiving a speaker’s articulatory mouth movements can alleviate the disadvantages in SiN comprehension that older adults experience due to sensory and cognitive aging to some extent. The phonological and temporal information provided by visible speech reduces the processing demands of speech and facilitates perception and comprehension (Peelle & Sommers, 2015; Sommers & Phelps, 2016). Accordingly, older and younger adults benefit from visible speech when perceiving SiN, both on a behavioral (e.g. Avivi-Reich et al., 2018; Smayda et al., 2016; Sommers et al., 2005; Stevenson et al., 2015; Tye-Murray et al., 2010; 2016) and on an electrophysiological level (Winneke & Phillips, 2011). The size of the benefit depends on the quality of the acoustic speech signal, or signal-to-noise ratio (SNR), as well as on individual auditory and visual perception and processing abilities (Tye-Murray et al., 2016). Once a certain noise threshold is reached, where individuals can no longer extract meaningful information from the auditory signal, they fail to exhibit any behavioral benefit from visible speech (Ross et al., 2007; Stevenson et al., 2015). As this threshold may be reached earlier in older than in younger adults due to age-related hearing loss, older adults may experience smaller visible speech benefits (e.g. Stevenson et al., 2015; Tye-Murray et al., 2010). Similarly, reduced lip-reading abilities in older adults may also lead to a smaller visible speech benefit (e.g. Sommers et al., 2005, Tye-Murray et al., 2010, 2016).

In addition to visible speech, the semantic information contained in iconic co-speech gestures also enhances speech comprehension and helps in the disambiguation of a lexically ambiguous or degraded speech signal, at least in younger adults. A large body of behavioral and neuroimaging research has shown that under optimal listening conditions, the information conveyed by iconic co-speech is integrated with speech during online language processing (e.g. Holle & Gunter, 2007; Kelly et al., 1999; 2010; Obermeier et al., 2011; for a review see Özyürek, 2014). For speech embedded in multitalker babble noise, word identification is better when sentences are accompanied by an iconic gesture (Holle et al., 2010) and listeners use iconic co-speech gestures to disambiguate lexically ambiguous sentences (Obermeier et al., 2012).

It is important to note that this previous research has investigated the effects of gestures in isolation, by blocking speakers’ heads or mouths from view. In every-day language use however, visible speech and co-speech gestures are not isolated phenomena, but naturally co-occur. Therefore, Drijvers and Özyürek (2017) and Drijvers et al. (2018) investigated the joint contribution of both visual articulators on word recognition in younger adults, using different levels of noise-vocoded speech.^1^ The combined effect of visible speech and gestures was significantly larger than the effect of either visual articulator individually, at least at a moderate noise vocoding level. At the worst vocoding level, where a phonological coupling of visible speech movements with the auditory signal was no longer possible (see also Ross et al., 2007; Stevenson et al., 2015), gestures provided the only source for a visual benefit.

Considering that iconic gestures provide such valuable semantic information to younger listeners under adverse listening conditions, one might expect their benefit to be comparable or even more pronounced for older adults, since older adults are more severely affected by SiN and have been shown to gain as much or more from additional semantic information (e.g. Pichora-Fuller et al., 1995; Smayda et al., 2016, for effects of sentence context on SiN comprehension).

However, there are indications that older adults may fail to process gestures in addition to speech, and/or to integrate gestures with speech. Cocks et al. (2011) found that older adults were just as good as younger adults in interpreting gestures without speech sound, i.e., visual-only presentation, but had difficulties interpreting co-speech gestures in relation to speech (note that here, the speaker’s face was covered, i.e. no information from visible speech was available). Under highly demanding listening conditions (i.e., very fast speech rates, dichotic shadowing), older adults similarly did not benefit from the semantic information contained in gestures in addition to visible speech, in contrast to younger adults (Thompson, 1995; Thompson & Guzman, 1999). Cocks et al. (2011, p. 34) suggest that it is possible that these findings are due to age-related WM limitations, as “the integration process [of speech and gesture] requires working memory capacity to retain and update intermediate results of the interpretation process for speech and gesture.” Older adults’ WM resources may have been consumed with speech processing operations, leaving insufficient resources for gesture comprehension and integration.

Therefore, as the ability to benefit from gestures may depend on an individual’s WM capacity, older adults may benefit less from gestures in addition to visible speech than younger adults, also when perceiving SiN. Furthermore, older adults may focus more strongly on the mouth area as a very reliable source of information, to the potential disadvantage of other sources of visual information (Thompson & Malloy, 2004), such that they might benefit less from gestures in the context of visible speech.

Since the contribution of visible speech and co-speech gestures to older adults’ processing of SiN has not been studied in a joint context, it is currently unknown whether older adults can benefit at all from the semantic information contained in co-speech gestures when perceiving SiN, in addition to the benefit derived from visible speech. Similarly, the role that changes in cognitive functioning associated with aging play in the processing of these multiple sources of visual information remains unknown. Given that both visible speech and iconic co-speech gesture form an integral part of human face-to-face communication, these articulators have to be considered jointly to gain a comprehensive and ecologically grounded understanding of older adults’ comprehension of SiN.

## The present study

The primary aim of the present study was therefore to investigate whether aging affects the comprehension of SiN perceived in the presence of visible speech and iconic co-speech gestures, and whether these processes are mediated by differences in sensory and cognitive abilities.

To explore this issue, we presented younger and older participants with a word recognition task in three visual contexts: speech-only (mouth blurred), visible speech, and visible speech + gesture. The speech signal was presented without background noise or embedded in two different levels of background multi-speaker babble noise, and participants had to select the written word they heard among a total of four words. These included a phonological as well as a semantic (i.e., gesture-related) distractor and an unrelated answer.

Generally, we expected that both age groups would perform worse at higher noise levels, and that older adults would be affected more strongly than younger adults, potentially mediated by hearing acuity. More importantly, we expected that younger adults’ word recognition in noise should improve most when both visual articulators (i.e. mouth movements and gesture) were present, as compared to the benefit from visible speech only, comparable to what has been found for younger adults using noise-vocoded speech (Drijvers & Özyürek, 2017; Drijvers et al., 2018). For the older adults, we refrained from making directed predictions on whether or not they, too, could make use of the semantic information contained in co-speech gesture in addition to visible speech, as the research summarized in the introductory section suggests that either outcome is conceivable (Cocks et al., 2011; Pichora-Fuller et al., 1995; Smayda et al., 2016; Thompson, 1995).

To test whether the expected differences between the two age groups in response accuracies and the size of the potential visual benefit is modulated by differences in cognitive abilities, we measured participants’ verbal and visual WM and inhibitory control. WM is assumed to be critical for online (language) processing, allowing for the temporary storage and manipulation of perceptual information (Baddeley & Hitch, 1974). Verbal WM capacity predicts comprehension and/or recall of SiN in older adults (Baum & Stevenson, 2017; Koeritzer et al., 2018; Rudner et al., 2016), potentially, because additional WM resources are recruited for the auditory processing of SiN, leaving fewer resources for subsequent language comprehension and recall. Visual WM capacity predicts gesture comprehension in younger adults, presumably playing a role in the ability to conceptually integrate the visuo-spatial information conveyed by gestures with the speech they accompany (Wu & Coulson, 2014). As the ability to process, update and integrate multiple streams of information may likewise depend on sufficient WM resources (Cocks et al., 2011), we expected higher WM capacities to be predictive of better performance overall, as well as a higher benefit of visible speech and gestures.

We additionally included a measure of inhibitory control, as the ability to selectively focus attention or to suppress irrelevant information has been connected to the comprehension of single talker speech presented against the background of several other talkers (i.e., multitalker babble, e.g. Janse, 2012; Jesse & Janse 2012; Tun et al., 2002). Therefore, we also expected better inhibitory control to be predictive of higher performance overall.

Finally, we evaluated the type of errors that participants made in the visible speech + gesture condition, to test whether older adults focus more exclusively on the mouth area than younger adults (Thompson & Malloy, 2004). If this were the case, we would expect them to make proportionally fewer gesture-based semantic errors and more visible speech-based phonological errors than younger adults in this condition.

## Method

### Participants

30 younger adults (14 women) between 20 and 26 years old (*M*_age_ = 22.04, *SD* = 1.79) and 28 older adults (14 women) between 60 and 80 years old (*M*_age_ = 69.36, *SD* = 4.68) took part in the study. The older participants were all community dwelling residents. The younger participants were students at Nijmegen University or Nijmegen University of Applied Sciences. All participants were recruited from the participant pool of the Max Planck Institute for Psycholinguistics and received between € 8 and € 12 for their participation, depending on the duration of the session. Participants were native Dutch speakers with self-reported normal or corrected-to-normal vision and no known neurological or language-related disorders. Educational level was assessed in terms of highest level of schooling. For the older participants, this ranged from secondary school level (25% of participants) via “technical & vocational training for 16 to 18-year-olds” (50% of participants) to university level (25% of participants). All of the younger participants were enrolled in a university program at the time of testing. The experiment was approved by the Ethics Commission for Behavioral Research from Radboud University Nijmegen. The data of two younger male participants were lost due to technical failure.

### Background measures

#### Hearing acuity

Hearing acuity was assessed with a portable Oscilla© USB-330 audiometer in a sound-attenuated booth. Individual hearing acuity was determined as the participants’ pure-tone average (PTA) hearing loss over the frequencies of ½, 1, and 2 kHz and 4 kHz. The data of one older male participant was lost due to technical failure. The average hearing loss in the older group was 24.95 dB (*SD* = 8.04 dB; Median = 22.5 dB; Range = 13.75 to 37.5 dB) and in the younger group 7.68 dB (*SD* = 3.58 dB; Median = 7.5 dB, Range = 0 to 15 dB). This difference was significant, Wilcoxon rank sum test, *W* = 4, *p* < 0.001.

#### Verbal WM

The backward digit-span task was used as a measure of verbal WM (Wechsler, 1981), which has been used in previous investigations of audiovisual processing and related topics in younger and older adults (e.g., Koch & Janse, 2016; Thompson & Guzman, 1999; Tun & Wingfield; 1999). Unlike word or listening/reading span tasks, the digit-span task has the advantage of not being affected by word semantics or frequency (Jones & Macken, 2015). Participants repeated digit sequences of increasing length in reverse order, requiring both item storage and manipulation (Bopp & Verhaeghen, 2005). Scores were computed as the longest correctly recalled sequence. Younger participants scored significantly higher than older participants, *M* = 5.21 (*SD* = 1.34; Median = 5; Range = 3 to 8) vs. *M* = 4.29 (*SD* = 1.24; Median = 4; Range = 0 to 7), *W* = 547, *p* = 0.009.

#### Visual WM

The Corsi Block-Tapping Task (CBT, Corsi, 1972) provides a measure of the visuo-sequential component of visual WM. Participants imitated the experimenter in tapping nine black cubes mounted on a black board in sequences of increasing length. Scores were calculated as the length of the last correctly repeated sequence multiplied by the number of correctly repeated sequences. Younger adults performed significantly better than older adults, *M* = 48.71 (*SD* = 19.74; Median = 42; Range = 30 to 126) vs. *M* = 25.71 (*SD* = 9.28; Median = 25; Range = 12 to 42), *W* = 721, *p* < 0.001.

#### Inhibitory control

Trail Making Test parts A and B (Parkington & Leiter, 1949) were used to assess inhibitory control. This test has been used in previous investigations of audiovisual processing in younger and older adults (e.g., Jesse & Janse, 2012; Smayda et al., 2016). In part A, participants connected circled numbers in sequential order. In part B, they alternated between numbers and letters, requiring the continuous shifting of attention. The difference between the times needed to complete both parts (i.e. B-A) provides a measure of inhibition/interference control, as it isolates the switching component of part B from the visual search and speed component of part A (Sanchez-Cubillo et al., 2009). The mean difference between parts B and A was significantly larger for the older adults M = 29.54 s (*SD* = 12.88; Median = 29; Range = 3.7 to 65) than for the younger adults *M* = 16.9 s (*SD* = 8.41; Median = 15.65; Range = 6 to 47.2), *W* = 142, *p* < 0.001.

### Pretest

We conducted a pretest to establish the noise levels at which younger and older adults might benefit most from perceiving gestural information in addition to visible speech (reported in detail in the supplementary materials, section A). Based on this pretest, we selected SNRs -18 and -24 dB for the main experiment.

### Materials

**Fig. 1:**
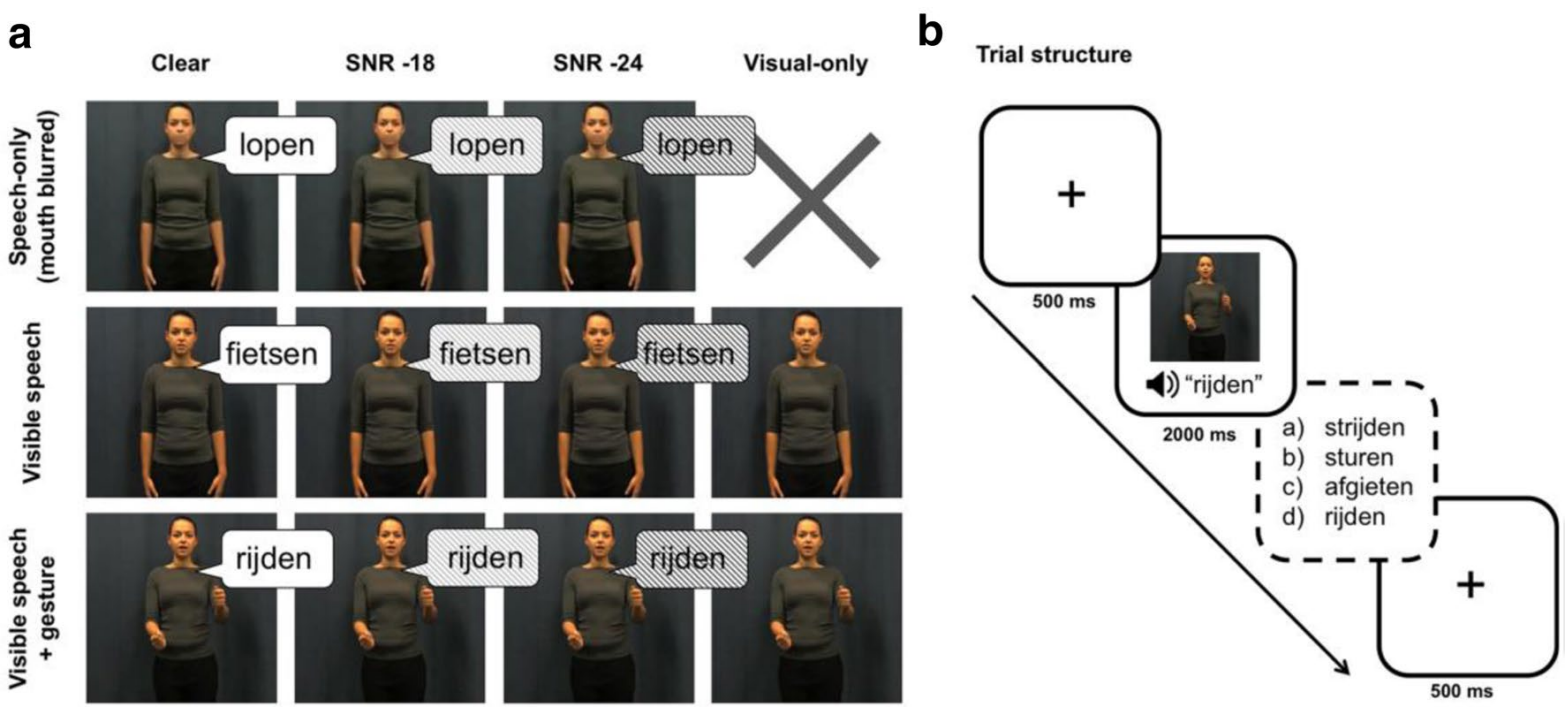
Experimental overview. **a** Overview of conditions. Action words are in Dutch: *lopen* (“to walk”), *fietsen* (“to cycle”), *rijden* (“to drive”). **b** Trial structure. Answer options are in Dutch: *strijden* (“to fight”, phonological competitor), *sturen* (“to steer”, semantic competitor), *afgieten* (“to drain”, unrelated foil), *rijden* (“to drive”, target)

The materials in this experiment were similar to the set of stimuli used in Drijvers & Özyürek (2017) and consisted of 220 videos of an actress uttering a highly frequent Dutch action verb while she was displayed with either having her mouth blurred, visible, or visible and accompanied by a co-speech gesture (see Fig. 1, panel A). All verbs were unique and only displayed in one condition. All gestures depicted the action denoted by the verb iconically, e.g. a steering gesture resembling the actress holding a steering wheel for the verb *rijden* (“to drive”). Gestures were matched on how well they fit with the verb, i.e. their iconicity (see Drijvers & Özyürek, 2017). Each video had a duration of 2 s, with an average speech onset of 680 ms after video onset. Gesture preparation started 120 ms after video onset, and the ‘stroke’, i.e. the most effortful and meaning-bearing part of the gesture (Kendon, 2004; McNeill, 1992), coincided with the spoken verb.

The speech in the videos was either presented as clear speech or embedded in eight-talker babble, with an SNR of -18, or with an SNR of -24. The babble was created by overlaying 20 s fragments of talk of eight speakers (four male and four female) using the software Praat (Boersma & Weenink, 2015). Subsequently, the babble was edited into 2 s fragments and merged with the original sound files using the software Audacity®. The background babble started as soon as the video started and commenced until the video was fully played. The sound of the original videos was intensity scaled to 65 dB. To create videos with SNR-18, the original sound file was overlaid with babble at 83 dB, for SNR-24 with babble at 89 dB.

To test for the contribution of gestures in addition to visible speech to the comprehension of SiN, we divided the 220 videos over 11 conditions, with 20 videos per condition (for a schematic representation see Fig. 1, panel A). Combining the three visual modalities (speech-only [mouth blurred], visible speech, visible speech + gesture) and three audio conditions (clear speech, SNR -18, SNR -24) yielded nine audiovisual conditions.^2^ Two additional conditions without audio were included to test how much information participants could obtain from visual-only information: no-audio + visible mouth movements, which is similar to assessing lip-reading ability, and no-audio + visible mouth movements + gesture, assessing people’s ability to grasp the semantic information conveyed by gestures in the presence of visible speech.

We created 28 experimental lists (each list was tested twice, once for a younger and once for an older participant). These lists were created by pseudo-randomizing the order of the 220 videos. Each participant saw each of the 220 videos exactly once in either of the four audio conditions; across the experiment, each video occurred equally often in each audio condition. Per list, the same audio or visual condition could not occur more than five times in a row.

The answer options contained four action verbs: (1) the target verb uttered by the actress; (2) a phonological competitor related to the target verb phonologically; (3) a semantic competitor related to the gesture (if present in the video); and (4) an unrelated foil (see Fig. 1, panel B). The semantic competitors were selected on the basis of a pretest (reported in Drijvers & Özyürek, 2017) and consist of action verbs that could plausibly be accompanied by the iconic gesture, i.e., the meaning of the gesture could be mapped to both the target and the competitor. Examples are a “driving” gesture (i.e., moving the hands as if holding a steering wheel) with the target “to drive” (*rijden*) and the semantic competitor “to steer” (*sturen*, see Fig. 1, panel B), or a “sawing” gesture (i.e., moving hand back and forth as if holding a saw) with the target verb “to saw” (*zagen*) and the semantic competitor “to cut” (*snijden*). The four answer options were presented in random order.

Due to a technical error in video presentation, one video had to be removed from the entire dataset, resulting in 219 trials per participant.

### Procedure

All participants received a written and verbal introduction to the experiment and gave their signed informed consent. For the main part of the experiment, participants were explicitly instructed to react as accurately and as quickly as possible.

First, hearing acuity was tested as described in Sect. 2.2. Subsequently, participants performed the main experiment, seated in a dimly lit sound proof booth and supplied with headphones. Videos were presented full screen on a 1650 × 1080 monitor using Presentation software (Neurobehavioral Systems, Inc.) with the participant at approximately 70 cm distance from the monitor. All trials started with a fixation cross of 500 ms, after which the video was played. Then the four answer options were displayed on the screen in writing, numbered a) through d). Participants chose their answer by pushing one of four accordingly numbered buttons on a button box (see Fig. 1, panel B for a schematic representation of the trial structure). After every 80 trials, participants could take self-timed breaks. Depending on the participant, this main part of the experiment took approximately 30 to 40 min. Afterwards, participants performed the cognitive tests as described above, and filled in a brief self-rating scale to assess their personal attitudes towards gesture production and comprehension (adapted from ‘Brief Assessment of Gesture’ (BAG) tool, Nagels, Kircher, Steines, Grosvald, & Straube, 2015) as well as a short questionnaire assessing how they made use of the gestures in the current experiment. Older adults agreed significantly less than younger adults with the statement “I like talking to people who gesture a lot while they talk” (*W* = 584, Bonferroni-adjusted *p* = 0.01), but did not significantly differ on any other item. In total, the experimental session lasted between 50 and 75 min, depending on the participant.

### Statistical methods

We performed three sets of analyses: one for response accuracies, one for the relative benefits of visible speech, of gestures, and of both combined, and one for the proportion of semantic and phonological errors in the visible speech + gesture condition. In line with previous literature on the benefit of visible speech on speech comprehension (e.g. Smayda et al., 2016; Stevenson et al., 2015), we focus our analyses on response accuracies rather than response latencies. However, we report the analyses of the response latencies in the supplementary materials (section B).

We conducted all analyses in the statistical software R (version 3.3.3, R Development Core Team, 2015), fitting (generalized) linear mixed effects models using the functions *glmer* and *lmer* from the package *lme4* (Bates et al., 2017).

Analyses were conducted in two steps: first, we evaluated only the experimental predictor variables, their interactions, and the mean-centered pure-tone averages (PTA) as a covariate, applying a backwards model-stripping procedure to arrive at the best-fitting models. We did this by removing interaction terms and predictor variables stepwise based on *p*-values, using likelihood-ratio tests for model comparisons. In a second step, we used these best-fitting models as a basis to which we added the mean-centered cognitive variables as covariates to test whether additional variation could be explained by differences in cognitive functioning.

All models contained by-participant random intercepts, but no by-item random intercepts, as not all items (i.e., verbs) occurred in all visual modalities. Also, we did not include by-participant random slopes for noise or visual conditions, as this led to convergence failures throughout.

Only the fixed effect estimates, standard errors of the estimates, and estimates of significance of the most parsimonious models are reported. Reported *p*-values were obtained via the package *lmerTest* (Kuznetsova et al., 2016). We used the function *glht* from the package *multcomp* (Hothorn et al., 2017) in combination with custom-built contrasts to explore individual contrasts where desired, correcting for multiple comparisons.

#### Response accuracies

We analyzed response accuracies as a binary outcome, scoring 0 for incorrect responses and 1 for correct responses.

#### Relative benefit

Additionally, we computed each participant’s relative benefit scores based on the average response accuracies for each multimodal condition, using the formula *(A – B)*/*(100 – B)* (Sumby & Pollack, 1954; Drijvers & Özyürek, 2017). This relative benefit allows for a direct comparison of how much older and younger adults benefitted from the different types of visual information. Additionally, it adjusts for the maximum gain possible and corrects for possible floor effects (see Sumby & Pollack, 1954; see also Ross et al., 2007, for a critical discussion of different benefit scores). The *visible speech benefit* was thus computed as *(visible speech – speech-only)/(100 – speech-only)*, the *gestural benefit* was computed as *(visible speech* + *gesture – visible speech)/(100 – visible speech)*, and the *double benefit* was computed as *(visible speech* + *gesture – speech-only)/(100 – speech-only)*.

In fitting the models predicting the relative benefit, we excluded data from “clear” trials, as performance for both age groups was near ceiling and participants often scored at perfect accuracy in the speech-only (mouth blurred) and visible speech conditions, which placed a zero in the denominator of the relative benefit formula.

#### Proportion of semantic and phonological errors

We computed the proportion of semantic and phonological errors out of all errors made in the visible speech + gestures condition. Rather than using raw error counts or proportion of errors out of all answers, these proportions of errors out of errors account for the possibility that one age group made more errors than the other across the board. Note that we excluded error proportion data for “clear” trials, as performance was frequently at perfect accuracy.

## Results

We first present the analyses of the response accuracies, followed by the analyses of the relative benefit of visible speech, gestures, and both combined, and the analyses of error proportions.

### Response accuracies

**Fig. 2:**
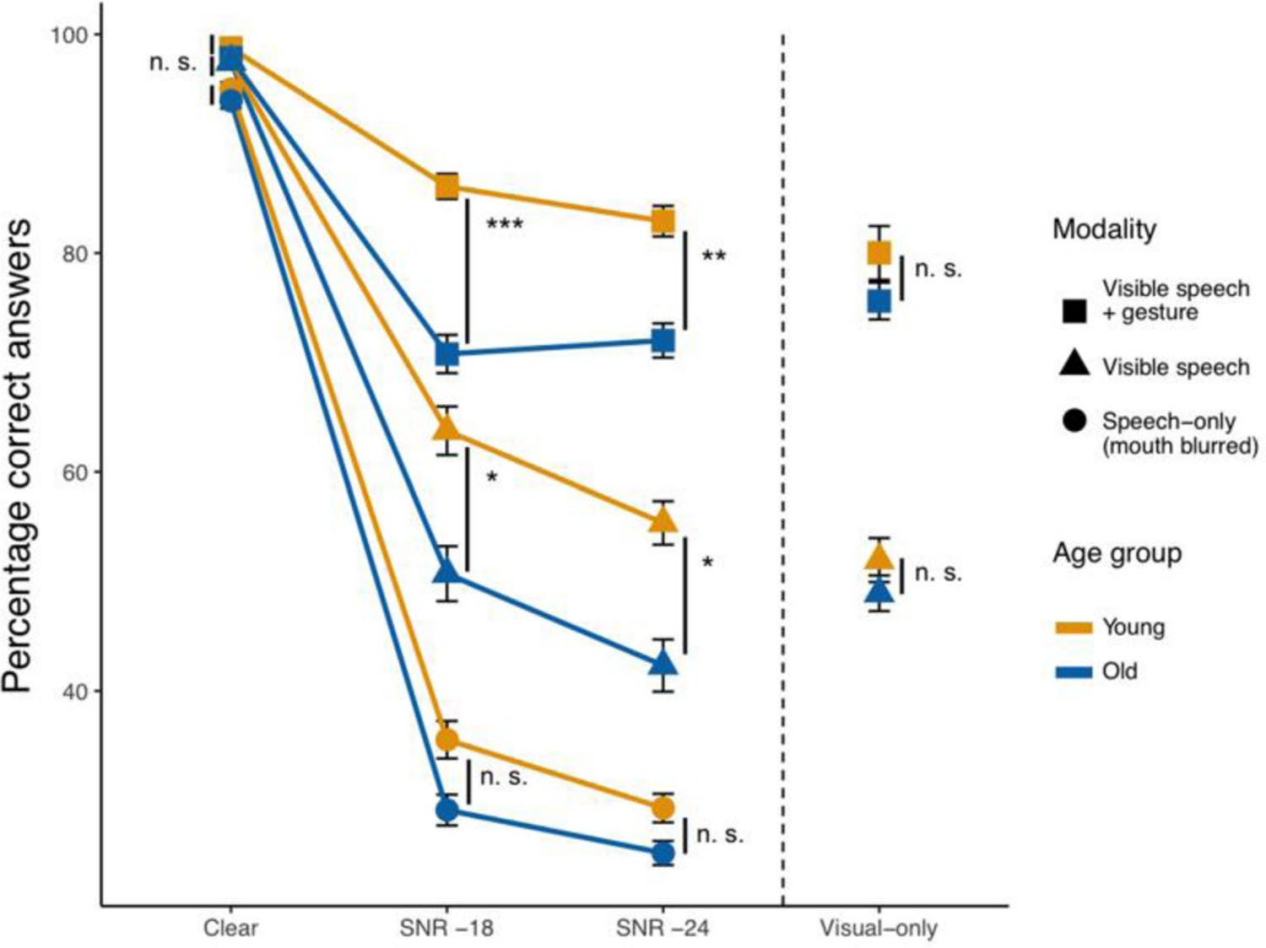
Response accuracy in percent per age group and condition. Error bars represent SE. The dotted line separates the audiovisual trials (left) from the visual-only trials (right). ***p < 0.001, **p < 0.01, *p < 0.05

Figure 2 represents the response accuracies in the audiovisual trials (i.e. the conditions with video and sound) and visual-only trials (i.e. the conditions with only video, no sound). Visual inspection of the data suggested that older adults did not perform better than chance in the speech-only, SNR-24 trials. A Wilcoxon signed rank test confirmed this (*V* = 97, *p* = 0.48). Since this concerns only one condition, we decided to conduct our analyses as planned. First, we compared response accuracies in the audiovisual trials based on age group and visual modality. In a second set of analyses, we followed up on the significant interaction of age by visual modality, analyzing audiovisual and visual-only trials separately per visual modality.

#### Audiovisual trials

An initial model predicting response accuracies in the audiovisual trials based on age group, visual modality, and noise failed to converge. As our main research question and predictions related to the factors age group and visual modality, we decided to include only these two factors in this first part of the analyses, collapsing across noise levels. The younger adults’ performance in the visible speech condition was used as a baseline level (intercept), to which we compared the older adults and other visual modality conditions. The best-fitting model (summarized in Table 1) shows significant effects for age and visual modality, such that younger adults outperformed older adults, while more visual articulators lead to higher accuracies. The significant interaction of the two factors indicates that the age-related performance difference was larger in the visible speech condition than in the speech-only condition, and again larger in the visible speech + gesture condition.^3^

**Table 1:** Model predicting response accuracy in multimodal trials, age group = young and visual modality = visible speech are on the intercept. *N* = 56

	*Response accuracy*			
	*β*	*SE*	*z*	*p*
Intercept	.97	.07	13.49	< .001
Age group_*old*_	– .40	.10	– 4.07	< .001
Visual modality_*Speech-only (mouth blurred)*_	–.83	.07	– 11.32	< .001
Visual modality_*Visible speech* + *gesture*_	1.17	.10	12.15	< .001
Age group_*old*_: Visual modality_*Speech-only (mouth blurred)*_	.25	.10	2.42	.02
Age group_*old*_: Visual modality_*Visible speech* + *gesture*_	–.32	.13	– 2.55	.01

Pairwise comparisons revealed that younger adults’ response accuracy was not higher than older adults’ in the speech-only (mouth blurred) condition (*β* = -0.16, *SE* = 0.10, *z* = -1.65, *p* = 0.45), but it was significantly higher in the visible speech condition (*β* = -0.40, *SE* = 0.10, *z* = -4.07, *p* < 0.001) and in the visible speech + gesture condition (*β* = -0.73, *SE* = 0.12, *z* = -6.04, *p* < 0.001). Furthermore, both age groups scored significantly higher in the visible speech condition than in the speech-only (mouth blurred) condition (YAs: *β* = 0.83, *SE* = 0.07, *z* = 11.32, *p* < 0.001; OAs: *β* = 0.59, *SE* = 0.07, *z* = 8.28, *p* < 0.001). Likewise, both age groups scored higher in visible speech + gesture condition than in the visible speech condition (YAs: *β* = 1.17, *SE* = 0.10, *z* = 12.15, *p* < 0.001; OAs: *β* = 0.85, *SE* = 0.08, *z* = 10.63, *p* < 0.001).

In summary, although both age groups performed better the more visual articulators were present, the age-related performance difference also increased as more visual information was present. Note that hearing acuity did not improve the model fit.

***Cognitive abilities in the audiovisual trials. ***Including the cognitive abilities yielded a significant effect of verbal WM, such that better WM was associated with higher accuracies (*β* = 0.11, *SE* = 0.04, *z* = 2.74, *p* = 0.006). The effect size of age group was reduced but remained significant (*β* = 0.32, *SE* = 0.10, *z* = -3.32, *p* < 0.001). Remaining effects or interactions were not affected.

#### Audiovisual and visual-only trials

To follow up on the significant interaction of age by visual modality and to be able to incorporate noise as a predictor in the analyses, we analyzed the audiovisual and, where applicable, visual-only trials separately per modality. Including the visual-only trials allowed us to investigate possible age differences in these conditions, and to draw direct comparisons between performance in visual-only and audiovisual trials.

***Speech-only (mouth blurred) trials.*** Within the speech-only (mouth blurred) trials, performance was best predicted by hearing acuity and noise, such that participants with better hearing acuity performed significantly better, while louder noise levels lead to worse performance (see Table 2). There was no significant effect for age group on response accuracy and no interaction with noise, indicating that younger and older adults’ performance did not differ significantly at any noise level (note though that the comparison between the two age groups at SNR-24 should be treated cautiously as the older adults’ chance level performance in this condition may be masking lower actual performance).

**Table 2:** Models predicting response accuracy in speech-only (mouth blurred), visible speech, and visible speech + gesture trials, age group = young and noise = SNR -18 are on the intercept. *N* = 56^a^

	*Speech-only (mouth blurred)*				*Visible speech*				*Visible speech* + *gesture*			
	*β*	*SE*	*z*	*p*	*β*	*SE*	*z*	*p*	*β*	*SE*	*z*	*p*
Intercept	– .75	.07	– 11.07	< .001	.59	.13	4.60	< .001	1.91	.15	12.40	< .001
Hearing acuity (PTA)	– .15	.05	– 3.12	.002	–	–	–	–	–	–	–	–
Age group_*old*_	–^b^	–	–	–	– .57	.18	– 3.13	.002	– .99	.20	– 4.93	< .001
Noise_*clear*_	3.64	.15	24.29	< .001	3.17	.29	11.08	< .001	2.57	.40	6.44	< .001
Noise_*SNR -24*_	– .24	.09	– 2.57	.01	– .37	.13	– 2.93	.003	– .25	.17	– 1.49	.14
Noise_*visual-only*_	n.a.^c^	n.a	n.a	n.a	– .51	.13	– 4.08	< .001	– .46	.16	– 2.78	.006
Age group_*old*_: Noise_*clear*_	–	–	–	–	.60	.41	1.48	.14	.41	.50	.82	.41
Age group_*old*_: Noise_*SNR -24*_	–	–	–	–	.01	.18	.04	.97	.32	.22	1.47	.14
Age group_*old*_: Noise_*visual-only*_	n.a	n.a	n.a	n.a	.43	.18	2.47	.01	.72	.21	3.33	< .001

^a^In the model predicting response accuracy in the speech-only (mouth blurred) condition, *N* = 55

^b^A hyphen indicates a non-significant predictor that was eliminated in the model-comparison process

^c^Note that there were no visual-only trials in the speech-only (mouth blurred) condition

*Cognitive abilities in the speech-only (mouth blurred) trials. *Verbal WM contributed significantly to the model fit (*β* = 0.13, *SE* = 0.05, *z* = 2.67, *p* = 0.008), reducing the size of the effect of hearing acuity (*β* = – 0.12, *SE* = 0.05, *z* = – 2.42, *p* = 0.02).

***Visible speech trials.*** Within the visible speech trials, older adults generally performed worse than younger adults, and both age groups performed worse at louder noise levels. The significant interaction of age group by noise indicates that the age-related performance difference was not equally large at all noise levels (Table 2). Pairwise comparisons revealed that younger and older adults differed from each other in their performance at SNRs -18 (*β* = -0.57, *SE* = 0.18, *z* = – 3.13, *p* = 0.02) and -24 (*β* = – 0.56, *SE* = 0.18, *z* = – 3.10, *p* = 0.02), but not in clear speech or in visual-only trials (both *p*’s > 0.5). Comparing the performance at the individual noise levels for the two age groups separately, we found that younger adults performed significantly better in SNR -18 than in SNR -24 and in visual-only trials (*β* = – 0.37, *SE* = 0.13, *z* = – 2.93, *p* = 0.03, and *β* = – 0.51, *SE* = 0.12, *z* = – 4.08, *p* < 0.001 respectively). There was no difference between SNR -24 and visual-only trials (*p* > 0.1). The older adults performed significantly better in SNR -18 than in SNR -24 (*β* = – 0.36, *SE* = 0.12, *z* = – 2.9, *p* = 0.03), but there were no differences between SNR -18 and visual-only trials, or between SNR -24 and visual-only trials (both *p*’s > 0.5). In summary, both age groups performed equally well in clear speech and visual-only trials, however, when background noise was added to the speech signal, younger adults significantly outperformed older adults. This was not related to differences in hearing acuity. Additionally, only for the younger adults, performance at the less severe noise level was better than in visual-only trials.

*Cognitive abilities in the visible speech trials. *Including verbal WM and inhibitory control improved the model fit (*β* = 0.14, *SE* = 0.07, *z* = 1.89, *p* = 0.059 and *β* = 0.18, *SE* = 08, *z* = 2.22, *p* = 0.03, respectively). This reduced the effect of age (*β* = – 0.29, *SE* = 0.19, *z* = – 1.49, *p* > 0.1), but did not affect other effects or interactions.

***Visible speech + gesture trials. ***Within visible speech + gesture trials, again, younger adults outperformed older adults, and louder noises lead to worse performance overall. As for visible speech, there was a significant interaction age group by noise (see Table 2). Pairwise comparisons revealed that younger and older adults differed from each other in their performance at SNRs -18 (*β* = – 0.99, *SE* = 0.20, *z* = – 4.93, *p* < 0.001) and -24 (*β* = – 0.68, *SE* = 0.20, *z* = – 3.45, *p* = 0.005), but not in clear speech or in visual-only trials (both *p*’s > 0.5). Comparing the performance at the individual noise levels for the two age groups separately, we found that younger adults performed significantly better at SNR -18 than in visual-only trials (*β* = – 0.46, *SE* = 0.16, *z* = – 2.78, *p* = 0.047), but there was no difference between SNRs -18 and -24 and between SNR -24 and visual-only (both *p*’s > 0.5). For older adults, there were no significant differences between SNRs -18 and -24, between SNR -18 and visual-only, or between SNR -24 and visual-only (all *p*’s > 0.5). Thus, as for visible speech, both age groups performed equally well in clear speech and in visual-only trials, but older adults performed significantly worse once background noise was added to the speech signal. Again, this was not related to hearing acuity. Additionally, only the younger adults performed better at the less severe noise level as compared to the visual-only trials.

*Cognitive abilities in the visible speech* + *gesture trials. *Including verbal WM significantly improved the model fit (*β* = 0.29, *SE* = 0.07, *z* = 4.12, *p* < 0.001). This reduced the effect size of age group without compromising its significant contribution as an explanatory variable (*β* = -0.79, *SE* = 0.19, *z* = -4.14, *p* < 0.001). Other effects or interactions were not affected.

### Relative benefit

**Fig. 3:**
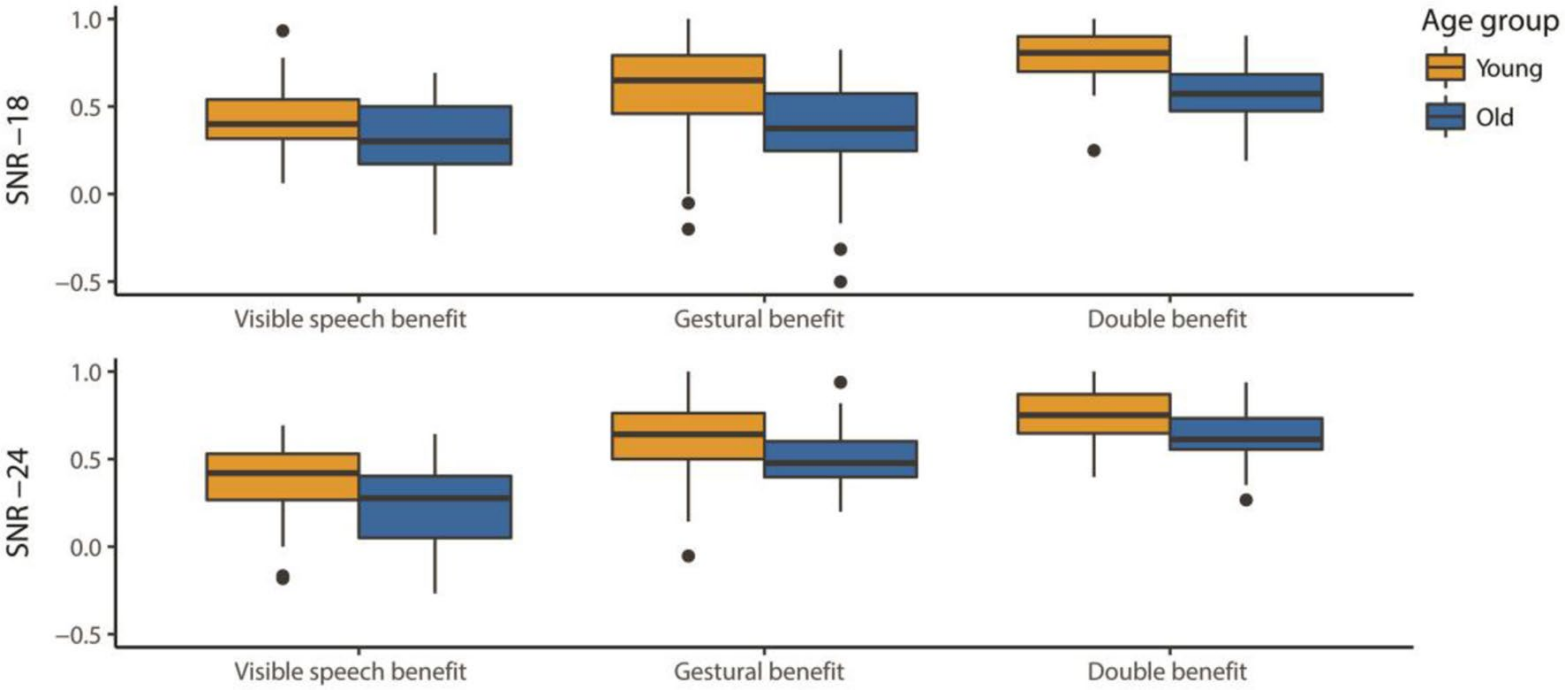
Relative benefit per age group, noise level, and benefit type. The black line represents the median; the two hinges represent the 1st and 3rd quartile; the whiskers capture the largest and smallest observation but extend no further than 1.5 * IQR (data points outside 1.5 * IQR are represented by dots)

The relative benefit indicates how much participants’ performance improves due to the presence of visible speech compared to speech-only (visible speech benefit), visible speech + gesture compared to visible speech (gestural benefit), or visible speech + gesture compared to speech-only (double benefit). The best-fitting model predicting the influence of age, noise, and benefit type on the size of the relative benefit is summarized in Table 3. The main effect of age shows that overall, older adults received a smaller benefit from visual information than younger adults. There was a significant interaction of benefit type by noise, but no interactions between age group and noise, or between age group and benefit type, suggesting that the pattern of enhancement was comparable for the two age groups (see also Fig. 3; note that we might be underestimating the size of the true benefits older adults received at SNR-24 due to their chance performance in the speech-only condition).

**Table 3:** Model predicting the size of the relative visual benefit, age group = young, benefit type = gestural benefit, and noise = SNR -18 are on the intercept. *N* = 56

	*Benefit size*			
	*Β*	*SE*	*t*	*p*
Intercept	.51	.04	14.28	< .001
Age group_*old*_	– .14	.03	– 4.50	< .001
Benefit type_*Visible speech*_	– .07	.04	– 1.53	.13
Benefit type_*Double*_	.24	.04	5.68	< .001
Noise_*SNR -24*_	.11	.04	2.67	.008
Benefit type_*Visible speech*_: Noise_*SNR -24*_	– .20	.06	– 3.23	.001
Benefit type_*Double*_: Noise_*SNR -24*_	– .11	.06	– 1.80	.07

We followed the significant interaction between benefit type and noise up by paired comparisons, to test whether the size of the individual benefit types changes from one noise level to the next. The visible speech benefit did not change from one noise level to the other (*p* > 0.10). The gestural benefit increased from SNR -18 to SNR -24; this approached significance (*β* = 0.11, *SE* = 0.04, *z* = 2.67, *p* = 0.057). The double benefit (i.e. the benefit of visible speech + gesture compared to speech-only [mouth blurred]) did not significantly change from one noise level to the other (both *p*’s > 0.1).

Subsequently, we compared the size of the individual benefits per noise level, to test whether the benefit of visible speech and gesture combined exceeds that of either articulator individually. At SNR -18, the size of the gestural benefit did not differ significantly from that of the visible speech benefit (*p* > 0.1). The double benefit was larger than both the gestural benefit (*β* = 0.24, *SE* = 0.04, *z* = 5.68, *p* < 0.001) and the visible speech benefit (*β* = 0.31, *SE* = 0.04, *z* = 7.21, *p* < 0.001). At SNR -24, the gestural benefit was larger than the benefit of visible speech (*β* = 0.26, *SE* = 0.04, *z* = 6.10, *p* < 0.001), and the double benefit was again larger than the gestural benefit (*β* = 0.13, *SE* = 0.04, *z* = 3.13, *p* = 0.01) and the visible speech benefit (*β* = 0.39, *SE* = 0.04, *z* = 9.29, *p* < 0.001).

Overall then, younger adults benefitted more from visual information than older adults. At the same time, both age groups received a larger benefit from both visual articulators combined than from each articulator individually at both noise levels. Note that neither hearing acuity nor cognitive abilities significantly contributed to the model fit.

### Proportion of semantic and phonological errors in visible speech + gesture trials

The best models predicting the proportion of semantic errors and of phonological errors both contained age group as the only significant predictor. Across all noise levels, older adults made a significantly higher proportion of semantic errors than younger adults (*β* = 10.45, *SE* = 5.03, *t* = 2.08, *p* = 0.043) and a significantly lower proportion of phonological errors (*β* = -9.29, *SE* = 3.95, *t* = -2.35, *p* = 0.02). For an overview of all answer types per age group and condition see supplementary materials, section C.

## Discussion

The present study provides novel evidence that younger and older adults benefit from visible speech and iconic co-speech gestures to varying degrees when comprehending speech-in-noise (SiN). This variation is partly accounted for by individual differences in verbal WM and inhibitory control, but could not be attributed to age-related differences in hearing acuity. Furthermore, the difference could also not be attributed to differences in the ability to interpret visual information (i.e., how well listeners understood gestures in the absence of speech). The individual results are discussed in more detail below.

Both younger and older adults benefitted from the presence of iconic co-speech gestures in addition to visible speech. For both age groups, response accuracies in the visible speech + gesture condition were higher than in the visible speech condition, and the relative benefit of both visual articulators combined was larger than the relative benefit of either only visible speech or only gestural information. Hence, younger and older adults were able to perceive and interpret the semantic information contained in co-speech gestures and to integrate it with the phonological information contained in visible speech.

Our results are in line with and extend Drijvers and Özyürek’s (2017) and Drijvers et al.’s (2018) findings on younger adults’ comprehension of a degraded speech signal to multitalker babble noise. At the same time, the present study is the first to show that older adults’ speech comprehension under adverse listening conditions, too, can benefit from the presence of iconic gestures. Earlier work on older adults’ SiN comprehension had mainly focused on the benefit of visible speech without taking gestures into account (e.g. Sommers et al., 2005; Stevenson et al., 2015; Tye-Murray et al., 2010; 2016). While these studies consistently report a benefit from visual speech, they do not allow for any conclusions with respect to the role of co-speech gestures, which are ubiquitous in every-day talk. We extend this body of work by showing that iconic co-speech gestures can provide an additional benefit on top of the benefit provided by visible speech.

In the light of our findings, it is important to note that work by Thompson (1995) and Thompson and Guzman (1999) suggested that older adults could not benefit from co-speech gestures in addition to visible speech under other highly challenging listening conditions, like speeded speech or dichotic shadowing. We suggest that the difference in findings between these previous studies and the present one is due to differences in task demands. The results of the present study show that in circumstances in which the effort of speech processing is comparatively low (single action verbs rather than sentences, no production component), older adults *are* able to make use of gestures in addition to visible speech to improve their comprehension of SiN. In the communication with older adults then, it might be useful to consider that the benefit from visual cues is potentially enhanced if the linguistic content is simplified or shortened.

Yet, the relative benefit that older adults received from visible speech, gestures, or both articulators combined was significantly smaller than the benefit that younger adults experienced. Although older adults’ chance performance in the more severe noise condition might mean that we underestimate their true ability to benefit from visual articulators at this noise level, the effects for the less severe noise level were reliable. Generally, our findings are in line with previous studies reporting a smaller benefit of visible speech for older adults under less favorable listening conditions (Stevenson et al., 2015; Tye-Murray et al., 2010). However, unlike reported in many previous studies on SiN, we did not find significant age-related performance differences in either of the unimodal conditions, i.e. the speech-only (mouth blurred) word recognition, or the visible speech and visible speech + gesture interpretation abilities (visual-only trials). Additionally, differences in hearing acuity did not predict performance in multimodal conditions or the size of the relative visual benefit. Therefore, in the present study, it seems unlikely that the age-related differences in response accuracies and in the relative visual benefit originated in age-related changes in hearing acuity, visual acuity, visual motion detection, or visual speech recognition. Yet, we would like to emphasize that based on our results, we do not make any claims as to whether visual-only speech recognition does or does not decrease in aging. It is possible that our design (using single action verbs, a cued recall task, and a small number of competitors) made the task relatively easier for older adults and therefore overestimates their true lip-reading ability. However, we feel confident to say that the age-related differences in the audiovisual conditions cannot be attributed to differences in visual-only speech recognition as it was assessed here.

Rather, age-related differences in the comprehension of SiN in the visible speech and visible speech + gesture conditions could at least in part be attributed to individual differences in verbal WM. In addition to that, individual differences in inhibitory control also predicted comprehension in the visible speech condition. This is in line with previous research on cognitive factors in SiN comprehension and visible speech (e.g. Baum & Stevenson, 2017; Rudner et al., 2016; Jesse and Janse, 2012; Tun et al., 2002). Our findings thus support the notion that due to the increased processing demands of the speech signal embedded in background talk, added WM and inhibitory resources are required for successful comprehension. Older adults were more strongly affected by the background noise than younger adults, presumably due to their relative decline in WM capacity and inhibitory control.

We therefore suggest that our findings reflect age-related changes in the processing of the auditory and visual streams of information during SiN comprehension. Younger adults used the visual information to enhance auditory comprehension where possible, resulting in higher response accuracies at the less severe noise level as compared to the visual-only trials. When the auditory signal was no longer at least minimally reliable at the more severe noise level, performance did not differ from the visual-only trials. This indicates that in more severe noise, visual information was the only valuable source of information (see also Drijvers & Özyürek, 2017).

For the older adults, on the other hand, performance in the audiovisual trials was not better than in the visual-only conditions. Potentially due to older adults’ limited verbal WM resources, which were additionally challenged by the increased processing demands of SiN, it was not possible to simultaneously attend to, comprehend, or integrate all sources of information (see also Cocks et al., 2011). Unlike in previous studies where older adults focused on the auditory signal (Cocks et al., 2011; Thompson, 1995; Thompson & Guzman, 1999), in the present study, they appeared to focus on the visual signal, presumably due to the greater reliability of the visual as opposed to the auditory signal.

Our interpretation is further supported by the trend for older adults to perform worse in audiovisual trials with background noise than in visual-only trials, that we did not observe for the younger adults. Myerson et al. (2016) similarly report cross-modal interference, such that unrelated background babble hinders younger and older adults’ ability to lip read (note however that Myerson et al. found no age difference in babble interference, but only in lip-reading ability). They suggest that either the monitoring of the speech stream left fewer resources for the processing of visual stimuli, or that the (attempted) integration of visual and auditory speech streams led to interference in the interpretation of the visible speech signal. This suggests that older adults may have spent more WM and inhibitory resources trying to comprehend, integrate, or suppress the background babble, subsequently lacking those resources for visual processing.

Although in principle, it is also conceivable that due to age-related hearing deficits, older adults received insufficient information from the auditory signal at both noise levels, making visual enhancement of the auditory signal impossible, we deem this an unlikely explanation. As we found no significant age-related performance difference in speech-only (mouth blurred) trials, and hearing acuity did not affect response accuracies in multimodal trials, we feel confident to assume that age-related hearing deficits cannot explain why younger adults were able to benefit from visible speech and gesture beyond the simple effect of visual information, but older adults were not.

In addition to age-related differences in hearing acuity, visible speech and gesture interpretation, and cognitive functioning, we also tested the possibility that older adults might pay more attention to visible speech than younger adults (Thompson & Malloy, 2004), to the potential detriment of gesture perception. However, we found that when co-speech gestures were available, older adults made more semantic (i.e. gesture-based) and fewer phonological (i.e. visible speech-based) errors than younger adults. This suggests that older adults actually focused *more* on gestural semantic information than on articulatory phonological information. In the present task, gestures presented a very reliable signal, and they may have been visually more accessible to older adults than visible speech due to the larger size of the manual as compared to the mouth movements.

Yet, it is important to note that older adults did not focus exclusively on the information contained in gestures, as the benefit of visible speech and gestures combined was larger than the individual benefit of either articulator, also for the older adults. Thus, multimodality enhances communication, despite age-related changes in cognitive abilities.

We are aware that the two noise levels employed in the present study may be considered relatively severe and potentially do not reflect the level of noise accompanying speech in most every-day contexts. The chance performance of older adults at the more severe noise level additionally limited our ability to draw strong conclusions about the true size of their visual benefit in this condition. Yet, the finding that older adults can benefit from visual information even under these conditions is novel and noteworthy in itself. Future research using less severe noise levels may show whether under these conditions, older adults’ ability to benefit from visible speech and gestures becomes more comparable to that of younger adults. Furthermore, we could only establish a gestural benefit for single words presented in isolation. Future research employing more complex linguistic material may show whether the beneficial effects of co-speech gestures also extend to longer stretches of speech.

## Conclusion

The present study provides novel insights into how aging affects the benefit from visible speech and from additional co-speech gestures during the comprehension of speech in multitalker babble noise. We demonstrated that when processing single words in SiN, older adults could benefit from seeing iconic gestures in addition to visible speech, albeit to a lesser extent than younger adults. Age-related performance differences were absent in unimodal conditions (speech-only or visual-only) and only emerged in multimodal conditions. Potentially, age-related working memory limitations prevented older adults from perceiving, processing, or integrating the multiple sources of information in the same way as younger adults did, thus leading to a smaller visual benefit. Yet, our findings highlight the importance of exploiting the full multimodal repertoire of language in the communication with older adults, who are often faced with speech comprehension difficulties, be it due to age-related hearing loss, cognitive changes, or background noise.

## Electronic supplementary material

Below is the link to the electronic supplementary material.

Supplementary file1 (DOCX 164 kb)

## Footnotes

1 Like Drijvers and Özyürek (2017), we use the term “visual articulators” to refer to both the articulatory movements of the mouth and manual co-speech gestures as the media via which information is conveyed, as this term is neutral with respect to intentionality.

2 Note that although labelled speech-only (mouth blurred) condition, participants may still glean some information from the speaker’s upper face in this condition, which may help identify SiN (Davis & Kim, 2006).

3 An alternative approach to addressing the convergence failure of the full model would have been to exclude the clear speech condition from the analysis, as both age groups performed near ceiling in this condition and variation was low. Analyzing this subset of the data yielded significant main effects for age group and visual modality and a significant interaction between age group and visual modality, nearly identical to those reported in the main body of the paper. Additionally, there was a main effect for noise, but no interactions between noise and the other predictors (either 2-way or 3-way). We nevertheless decided to report the analysis of the full dataset in the body of the paper, because including the clear speech condition is theoretically relevant and necessary to exclude the possibility that older adults perform worse than younger adults under optimal listening conditions, particularly in subsequent analyses.
