# Supplementary material for: Aging and working memory modulate the ability to benefit from visible speech and iconic gestures during speech-in-noise comprehension
Source: Psychol Res. 2020 Jul 5;85(5):1997–2011. doi: 10.1007/s00426-020-01363-8 (PMC8289811; doi:10.1007/s00426-020-01363-8)
Supplement: Supplementary file 1 — Supplementary file1 (DOCX 164 kb) [file 426_2020_1363_MOESM1_ESM.docx]

1. **Pretest**

10 younger adults (4 female), aged 22 to 30, and 10 older adults (9 female), aged 62 to 78, participated in the pretest. None of the participants from the pretest participated in the main experiment.

The task participants performed was identical to the task in the main experiment. Videos were presented with audio in either clear speech, or embedded in 8-talker babble at SNRs -6, -12, -18, or -24 dB. There were two multimodal conditions (speech + visible speech, speech + visible speech + gesture). For a detailed description of the materials, see section 2.4 of the main paper.

We performed logistic regression analyses for both age groups separately using the function *glmer* from the package *lme4* in the statistical software R, as described in the main paper, section 2.6. The contrasts between individual noise levels reported below were obtained using the package *lsmeans* (Lenth, 2017).

Both younger and older adults performed at or above 85% accuracy in the visible speech condition in SNRs -6 and -12, with no significant performance difference between the visible speech and the visible speech + gesture conditions (younger adults: SNR -6, *β* = .48, *SE* = .5, *z* = .97, *p* = .33; SNR -12, *β* = -.58, *SE* = .42, *z* = -1.4, *p* = .16; older adults: SNR -6, *β* = 7.2e-01, *SE* = .62, *z* = 1.16, *p* = .25; SNR -12, *β* = -1.11e-01, *SE* = .33, *z* = -.33, *p* = .74; see also Figure 1). For the younger adults, as performance decreased in the visible speech condition, the added value of gestures became significant at SNRs -18 (*β* = -1.05, *SE* = .3, *z* = -3.53, *p* < .001) and -24 (*β* = -1.5, *SE* = .26, *z* = -5.69, *p* < .0001). For older adults, performance similarly decreased with increasing noise in the visible speech condition, however, the difference between visible speech and visible speech + gesture remained non-significant at SNR -18 (*β* = -3.64e-01, *SE* = .25, *z* = -1.47, *p* = .14) and became significant only at SNR -24 (*β* = -1.07e, *SE* = .24, *z* = -4.45, *p* < .0001).

Figure 1. Response accuracies in percent per noise level and visual modality. Error bars represent SE.

**B. Response latencies**

We evaluated the effects of age group, visual modality, and noise on the log transformed response latencies of the correct trials by fitting a linear mixed effects model, using the function *lmer* from the package *lme4* in the statistical software R, as described in the main paper, section 2.6. The best-fitting model contained a significant three-way interaction of the predictors (the likelihood-ratio test was significant at *p* < .001 for comparing models with and without the three-way interaction term). In order to explore this three-way interaction further, we analyzed the response latencies of older and younger adults separately.

For the younger adults, more visual articulators led to shorter response latencies, and more severe noise led to longer response latencies (see Table 1, also Figure 2). The (non-)significant interactions of noise by modality indicate that the differences in response latencies between the three modalities were significantly larger at SNR -18 than at clear but did not differ between SNRs -18 and -24.

For the older adults, we found significant main effects of noise and of modality, but no interaction of the two predictors (see Table 2, also Figure 2). As for the younger adults, more severe noise led to longer response latencies. Response latencies were shorter in the visible speech + gesture trials than in the visible speech trials, but there was no difference between visible speech and speech-only trials.


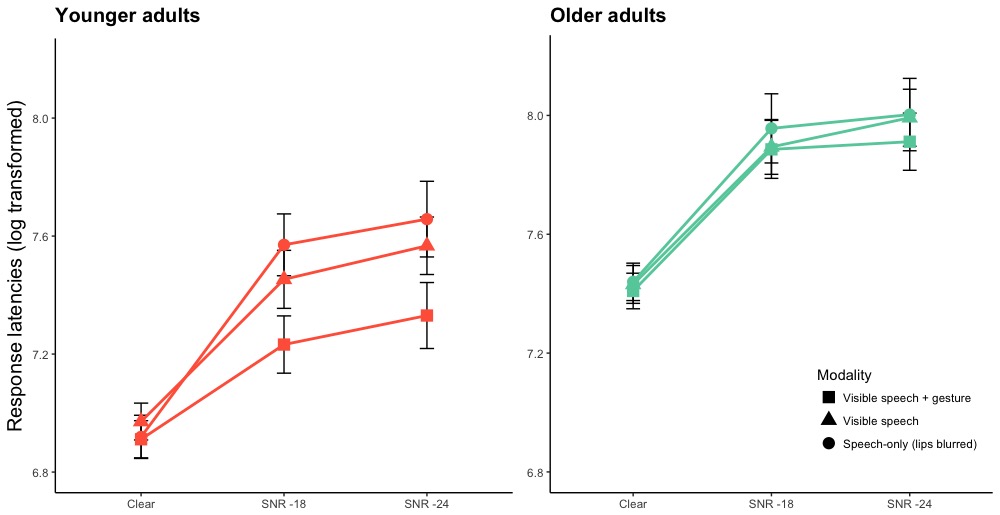


Figure 2. Log transformed response latencies per noise level and visual modality. Error bars represent SE.

**Table 2. Models predicting log transformed response latencies for younger and older adults separately. Noise = SNR -18 and modality = visible speech are on the intercept.**

|  | *Younger adults* | | | | *Older adults* | | | |
| --- | --- | --- | --- | --- | --- | --- | --- | --- |
|  | β | *SE* | *t* | *p* | β | *SE* | *t* | *p* |
| Intercept | 7.44 | .05 | 152.64 | <.001 | 7.92 | .04 | 207.62 | < .001 |
| Noise*_clear_* | -.48 | .03 | -16.95 | < .001 | -.49 | .02 | -28.05 | < .001 |
| Noise*_SNR -24_* | .13 | .03 | 3.86 | < .001 | .05 | .02 | 2.39 | .02 |
| Visual modality*_Speech-only (mouth blurred)_* | .11 | .04 | 3.04 | .002 | .02 | .02 | .90 | .37 |
| Visual modality*_Visible speech + gesture_* | -.21 | .03 | -7.20 | < .001 | -.04 | .02 | -2.29 | .02 |
| Noise*_clear_* : Visual modality*_Speech-only (mouth blurred)_* | -.16 | .04 | -3.57 | < .001 | - | - | - | - |
| Noise*_SNR -24_* : Visual modality*_Speech-only (mouth blurred)_* | .02 | .05 | -.44 | .66 | - | - | - | - |
| Noise*_clear_* : Visual modality*_Visible speech + gesture_* | .15 | .04 | 4.02 | < .001 | - | - | - | - |
| Noise*_SNR -24_* : Visual modality*_Visible speech + gesture_* | -.04 | .04 | -.84 | .40 | - | - | - | - |

^1^ A hyphen indicates a non-significant predictor that was eliminated in the model-comparison process.

**C. Average responses per answer type in percent (SD) per age group and condition.**

|  |  | *Young* | | | | *Old* | | | |
| --- | --- | --- | --- | --- | --- | --- | --- | --- | --- |
| *Modality* | *Noise* | *Target* | *Semantic competitor* | *Phonological competitor* | *Unrelated foil* | *Target* | *Semantic competitor* | *Phonological competitor* | *Unrelated foil* |
| *Speech-only (mouth blurred)* | *clear* | 95.00 (4.71) | 0.36 (1.31) | 1.43 (3.56) | 3.21 (2.44) | 93.93 (5.33) | 0.00 (0.00) | 2.32 (4.19) | 3.75 (2.59) |
|  | *SNR-18* | 35.54 (12.79) | 20.36 (8.60) | 24.46 (8.85) | 19.64 (9.71) | 29.11 (10.55) | 24.64 (9.71) | 25.54 (9.94) | 20.71 (9.88) |
|  | *SNR-24* | 29.29 (9.79) | 25.18 (10.93) | 22.14 (7.38) | 23.39 (10.37) | 25.18 (8.22) | 23.39 (10.98) | 27.68 (10.14) | 23.75 (10.15) |
| *Visible speech* | *clear* | 97.50 (5.53) | 0.18 (0.94) | 2.14 (5.52) | 0.18 (0.94) | 97.50 (3.19) | 0.36 (1.31) | 2.14 (2.86) | 0.00 (0.00) |
|  | *SNR-18* | 63.75 (16.59) | 7.32 (8.33) | 21.79 (12.56) | 6.79 (6.27) | 50.71 (18.79) | 15.89 (8.50) | 21.07 (9.16) | 11.79 (10.65) |
|  | *SNR-24* | 55.36 (14.84) | 12.86 (11.50) | 23.39 (7.94) | 8.39 (6.95) | 42.32 (17.87) | 18.04 (9.46) | 23.39 (11.39) | 15.89 (12.25) |
|  | *visual-only* | 51.96 (15.05) | 10.18 (8.55) | 24.11 (9.82) | 13.75 (10.24) | 48.93 (12.20) | 14.46 (8.20) | 23.21 (9.74) | 13.39 (12.70) |
| *Visible speech + gesture* | *clear* | 98.75 (2.93) | 0.36 (1.31) | 0.89 (2.38) | 0.00 (0.00) | 97.85 (3.46) | 0.89 (1.95) | 0.90 (1.97) | 0.36 (1.31) |
|  | *SNR-18* | 86.09 (8.62) | 8.33 (7.16) | 3.41 (3.07) | 2.17 (3.20) | 70.77 (13.09) | 21.67 (9.32) | 4.14 (5.80) | 3.42 (4.35) |
|  | *SNR-24* | 82.91 (10.46) | 10.33 (6.51) | 3.27 (4.42) | 3.49 (4.28) | 72.00 (11.71) | 20.23 (9.20) | 3.05 (4.16) | 4.72 (4.96) |
|  | *visual-only* | 80.01 (18.40) | 11.90 (8.08) | 4.30 (6.77) | 3.79 (6.90) | 75.61 (12.61) | 16.59 (8.47) | 3.26 (4.20) | 4.54 (5.06) |
